# Bulk RKKY signatures of topological phase transition in silicene

**DOI:** 10.1038/s41598-018-24567-w

**Published:** 2018-04-18

**Authors:** Hou-Jian Duan, Chen Wang, Shi-Han Zheng, Rui-Qiang Wang, Da-Ru Pan, Mou Yang

**Affiliations:** 1 Guangdong Provincial Key Laboratory of Quantum Engineering and Quantum Materials, School of Physics and Telecommunication Engineering, South China Normal University, Guangzhou, 510006 China; 2 School of Information Engineering, Guangdong University of Technology, Guangzhou, 510006 China

## Abstract

Silicene offers an ideal platform for exploring the phase transition due to strong spin-orbit interaction and its unique structure with strong tunability. With applied electric field and circularly polarized light, silicone is predicted to exhibit rich phases. We propose that these intricate phase transitions can be detected by measuring the bulk Ruderman-Kittel-Kasuya-Yosida (RKKY) interaction. We have in detail analyzed the dependence of RKKY interaction on phase parameters for different impurity configurations along zigzag direction. Importantly, we present an interesting comparison between different terms of RKKY interaction with phase diagram. It is found that the in-plane and out-of-plane terms can exhibit the local extreme value or change of sign at the phase critical point and remarkable difference in magnitude for different phase regions. Consequently, the magnetic measurement provides unambiguous signatures to identify various types of phase transition simultaneously, which can be carried out with present technique.

## Introduction

Topological quantum phase transition has received great interest in condensed matter of states for searching for new matter states^1^, such as very recently emerging topological insulators (TIs), Weyl or Dirac semimetals. Topological quantum state possesses many exotic and robust properties with potential application in quantum calculations^2,3^. Topological phases are usually classified with topological indices. In 2D quantum system, the topological indices are reduced to the charge- and spin-Chern numbers^2^, obtained by summation over the Berry curvature. Nevertheless, how to identify these different topological states experimentally is a challenging problem. The most instinctive method to detect a topological phase is to measure the spin-resolved quantum Hall conductivity or to directly probe topological states. However, these electric measurements are difficult to perform in quantum Hall systems and moreover topological edge states are easy to suffer from the disturbance from bulk states which are unavoidable due to the existence of imperfections in the composition.

Much effort is made to find other new tools for probing the topological phase transition. The phase-dependent heat currents provide a robust tool to distinguish the existence of topological Andreev bound states from trivial Andreev bound states in superconductor/TI Josephson junction^4^. To explore the existence of fractional quantum Hall states in TIs, authors^5^ presented thermoelectric measurements on the Bi_2_Te_3_ crystal. The magnetic susceptibility of electrons was studied in topological nodal semimetals, in which a giant anomaly is regarded to be useful in experimental identification of the Weyl, Dirac and line node semimetals^6^. The spin response in HgTe quantum wells^7^ reveals that unconventional spin-related properties can distinguish the paradigmatic TI material from the other 2D electronic systems.

Silicene, a single layer of silicon atoms with a planar honeycomb lattice structure^8–10^, offers an ideal platform for exploring the phase transition. Besides large spin-orbit interaction up to 3.9 meV^11^, silicene possesses a buckled hexagonal structure, in which two atoms in the translational unit cell reside on different planes, making its bandgap tunable easily by applying an electric field perpendicular to the silicene sheet^12^. The electric field breaks inversion symmetry while the circularly polarized light breaks time-reversal symmetry, both of which modify the Berry curvatures in the momentum space so that the occupied electronic states change the topological properties. When both of fields are applied, the silicene is predicted to exhibit rich phases: quantum spin Hall insulator (QSHI), conventional bulk insulator (CBI), photoinduced quantum Hall insulator (P-QHI), and photoinduced spin-polarized quantum Hall insulator (PS-QHI)^13^. It is an intriguing problem how to detect experimentally which phase the system stays in just by the bulk property. To probe these intricate phase transitions, Jin *et al*.^14^ have suggested to measure the Nernst conductivity, from which phase boundaries can be determined by comparison the charge- with spin-Nernst conductivities.

The Ruderman-Kittel-Kasuya-Yosida (RKKY) interaction, which describes the indirect exchange coupling between magnetic impurities mediated by the itinerant electrons, greatly depends on the spin-orbit interaction of host materials^15^. Meanwhile, the spin-orbit interaction plays a vital role in topological phase transitions. Thus, it is natural to expect that there is a close relation between the RKKY interaction and phase transition. For the phase transition from QSHI to CBI induced by electric field, Zare *et al*.^16^ found that the RKKY interaction can be used to identify the topological phase since its RKKY interaction is about 20 times greater than in the band insulator region when impurities are located on the edge. However, no difference in the order of magnitude appears in the case where the magnetic impurities are in the bulk. In this paper, we extend this study to more intricate phase transitions when silicene is subjected to both a circularly polarized light and a perpendicular electric field, where four types of phase are involved: QSHI, CBI, P-QHI, and PS-QHI. We have in detail analyzed dependence on phase parameters of RKKY interaction and present a RKKY phase diagram. It is interesting to find that the RKKY measurement provides unambiguous signatures to identify different phases and phase boundaries simultaneously. Moreover, all signatures originate from the bulk band and thus one can probe the topological phases only by measuring the bulk states, not caring for the formation of topological states.

## Model and Method

Silicene has a honeycomb lattice with two different atoms in the translational unit cell. Employing the tight-binding model for the four bands^13^, the Hamiltonian is given by^16^

1 $$H=-\,t\,\sum _{\langle i,j\rangle s}\,{c}_{is}^{+}{c}_{js}+i\frac{{\lambda }_{so}}{3\sqrt{3}}\sum _{\langle \langle i,j\rangle \rangle ss^{\prime} }\,{c}_{is}^{+}{\sigma }_{ss^{\prime} }\cdot ({{\bf{d}}}_{i}\times {{\bf{d}}}_{j}){c}_{js^{\prime} }+U\,\sum _{is}\,{\mu }_{i}{c}_{is}^{+}{c}_{is}$$

where 〈*i*, *j*〉 (〈〈*i*, *j*〉〉) runs over the nearest-neighbor (next-nearest-neighbor) hopping sites, ${c}_{is}^{+}$ creates an electron with spin *s* at site *i*, *σ* is the Pauli matrix of spin, **d**_*i*_ and **d**_*j*_ are the in-plane unit vectors along which the electron traverses from site *j* to *i*. The first two terms describe the silicene with hopping energy *t* = 1.6 eV and the intrinsic spin-orbit coupling *λ*_*so*_ ≈ 3.9 meV^11,17,18^, while the weak Rashba spin-orbital interaction is neglected^13^. The third term stands for the staggered potential with *μ*_*i*_ = ±1 for *A* (*B*) site and *U* = *E*_*z*_*d*/2, caused by an electric filed *E*_*z*_ exerting on the buckled lattice structure^19–21^, where two sublattice planes are separated by a distance of *d* = 0.46*Å*. By transforming Eq. (1) into the momentum space and then expanding it at the two Dirac points **K**_*η*_ (*η* = ±) in the Brillouin zone (BZ), we in the pseudospin space {*A*, *B*} obtain the low-energy Dirac Hamiltonian^16^

2 $${H}_{\eta s}=(\begin{array}{cc}{U}_{\eta s} & \hslash {v}_{F}k{{\rm{\Phi }}}_{{K}_{\eta }}\\ \hslash {v}_{F}k{{\rm{\Phi }}}_{{K}_{\eta }}^{\ast } & -{U}_{\eta s}\end{array}).$$

Here, ${v}_{F}=\frac{\sqrt{3}}{2}at$, *U*_*ηs*_ = −*sλ*_*so*_*η* − *U* with *s*,*η* = ±1 are the spin and valley indices, respectively, and ${{\rm{\Phi }}}_{{K}_{\eta }}=\eta {e}^{-i\pi \mathrm{/3}+i\eta \theta }$ with the polar angle *θ* = arctan (*k*_*y*_/*k*_*x*_) and an extra phase factor^22^ stemming from the specific *K*_*η*_.

In order to present rich phases, we assume the silicene sheet is in addition irradiated by a beam of circularly polarized light. The photoinduced effect is considered by the Peierls substitution *ħ***k** → *ħ***k** + *e***A**(*t*), where vector potential **A**(*t*) = *A*(sin *ωt*, cos *ωt*) is a periodic function of time *T* = 2*π*/*ω* with *ω* being the light frequency. By using the Floquet theory^13,23–27^, the time dependence can be mapped to a Hilbert space of time-independent multi-photon Hamiltonian. For the off-resonant light with the high-frequency limit ${A}^{2}/\omega \ll 1$, one can decouple the zero-photon state from the other states and only consider its dressed effect through second-order virtual photon absorption and emission processes^13,25,28,29^. As a consequence, the modified part of Hamiltonian by light reads *V*_*n*_ = [*V*_−1_, *V*_+1_]/*ħω* + *O*(*A*^4^) with ${V}_{n}=\frac{1}{T}\,{\int }_{0}^{T}\,H(t){e}^{-in\hslash \omega t}dt$ and the effective Hamiltonian is approximately expressed as

3 $${H^{\prime} }_{\eta s}={H}_{\eta s}+{V}_{n=0}={H}_{\eta s}+\eta {\rm{\Omega }}{\sigma }_{z},$$

with the illumination parameter ${\rm{\Omega }}=\frac{3{t}^{2}{A}^{2}}{4\hslash \omega }$. By diagonalizing the Hamiltonian ${H^{\prime} }_{\eta s}$, the low-energy dispersion reads

4 $${E}_{\eta s}^{\pm }=\pm \,\sqrt{{\hslash }^{2}{v}_{F}^{2}{k}^{2}+{U}_{\eta s}^{2}}$$

where the energy gap $2|{U}_{\eta s}|=2|({\rm{\Omega }}-s{\lambda }_{so})\eta -U|$ can be opened or closed, controlled by both the light and electric fields. Consequently, the topological phase transition occurs among four categories^13^: P-QHI, QSHI, PS-QHI, and CBI.

We assume two magnetic impurities **S**_*i*_ placed on the lattice sheet interacting with conducting electrons via ${H}_{int}=\lambda \,{\sum }_{i}\,{\bf{S}}({{\bf{r}}}_{i})\cdot {\bf{s}}({{\bf{r}}}_{i})$, where **S**(**r**_*i*_) [**s**(**r**_*i*_)] is the spin of impurities (itinerant electrons) and *λ* is the spin-exchange coupling strength. For weak coupling, we can replace *H*_*int*_ with the RKKY interaction, which in the second-order perturbation theory^15,30–33^ is given by

5 $${H}_{RKKY}^{\alpha \beta }=\frac{-{\lambda }^{2}}{\pi }\,{\rm{Im}}\,{\int }_{-\infty }^{{E}_{F}}\,{\rm{Tr}}[({{\bf{S}}}_{1}\cdot \sigma ){G}_{\alpha \beta }({\bf{R}},\varepsilon )({{\bf{S}}}_{2}\cdot \sigma ){G}_{\beta \alpha }(\,-\,{\bf{R}},\varepsilon )]d\varepsilon .$$

Here, *α*, *β* = {*A*, *B*}, **R** is spatial distance between two impurities, *E*_*F*_ is Fermi level, and the trace is over the spin degree of freedom. The retarded Green’s function ${G}_{\alpha \beta }({\bf{R}},\varepsilon )={\sum }_{\eta }\,\int \,{e}^{i({\bf{k}}+{{\bf{K}}}_{\eta }){\bf{R}}}{d}^{2}{\bf{k}}\,{[\mathrm{1/(}\varepsilon +i{0}^{+}-{H^{\prime} }_{\eta s})]}_{\alpha \beta }$ is a 2 × 2 matrix in spin space. In next discussions, we focus on the impurities placed on the same sublattice (e.g., *α* = *β* = *A*) and drop the subscript for briefness. Consequently, the matrix element of Green’s function is diagonal in spin space and reads

6 $${G}^{s}({\bf{R}},\varepsilon )=-\,\frac{2\pi }{\varsigma {\hslash }^{2}{v}_{F}^{2}}\,\sum _{\eta =\pm \,1}\,{e}^{i{{\bf{K}}}_{\eta }{\bf{R}}}(\varepsilon +{U}_{\eta s}){K}_{0}({ {\mathcal R} }_{{U}_{\eta s}}),$$

where *K*_0_(*x*) is the modified Bessel function of the second kind, *ς* is the area of BZ, and ${ {\mathcal R} }_{x}=R\sqrt{{x}^{2}-{\varepsilon }^{2}}/\hslash {v}_{F}$ with *R* = |**R**|. By inserting the above Green’s functions in Eq. (5), the RKKY interaction can be rewritten as^16^

7 $${H}_{RKKY}={J}_{\parallel }\,\sum _{i=x,y}\,{S}_{1i}{S}_{2i}+{J}_{z}{S}_{1z}{S}_{2z}+{J}_{DM}{({{\bf{S}}}_{1}\times {{\bf{S}}}_{2})}_{z},$$

which is divided into three terms according to the polarizations of the impurities.

## Numerical Results and Discussion

### RKKY under light field

To detect the topological phases, we expect to search for signatures of the RKKY interaction characterizing the phase-transition point and various phase regions. Firstly, we consider the case of silicene sheet irradiated by a beam of off-resonant light but in the absence of electric field. The light field breaks the time-reversal symmetry and so causes spin splitting |Ω ± *λ*_*so*_| in the energy spectrum from the original spin-degenerate bands *s* = ±1. With the increase of light strength, the bandgap is closed first at the critical point Ω = ±*λ*_*so*_ and then enters a new topological phase of P-QHI from QSHI state. Different topological phases can be classified by topological quantum numbers (*C*, *C*_*s*_), corresponding to charge- and spin-Chern numbers, respectively. They are usually defined as $C={\sum }_{\eta }\,({C}_{\uparrow }^{\eta }+{C}_{\downarrow }^{\eta })$ and ${C}_{s}={\sum }_{\eta }\,({C}_{\uparrow }^{\eta }-{C}_{\downarrow }^{\eta })/2$, and are calculated with the integral of a closed path ${C}_{s}=\frac{1}{2\pi }\,\sum _{n}\,{\int }_{BZ}\,d{\bf{k}}{{\rm{\Omega }}}_{xy}^{n}({\bf{k}})$ over the Berry curvature Ω^*n*^(**k**) of the *n*-th band^34^. Proceeding the calculation in silicene, we find that the topological index with different spins and valleys is ${C}_{s}^{\eta }=\frac{\eta }{2}{\rm{sgn}}(U-\eta {\rm{\Omega }}+s\eta {\lambda }_{so})$. Thus, according to the relative value of external fields, we can differentiate phase regions with (*C*, *C*_*s*_). For example, in the PS-QHI state, which is located in the area of |Ω − *U*| < *λ*_*so*_ and |Ω + *U*| > *λ*_*so*_, the topological index is found to be ${C}_{\uparrow /\downarrow }^{-}=-\,\mathrm{1/2}$ in valley **K**_−_ and ${C}_{\uparrow }^{+}=\mathrm{1/2}$ (${C}_{\downarrow }^{+}=-\,\mathrm{1/2}$) in valley **K**_+_, and so (*C*, *C*_*s*_) = (−1, 1/2). In the same way, the topological quantum numbers in other phase regions are calculated as: CBI with (0, 0), QSHI with (0, 1), P-QHI with (−2, 0).

**Figure 1:**
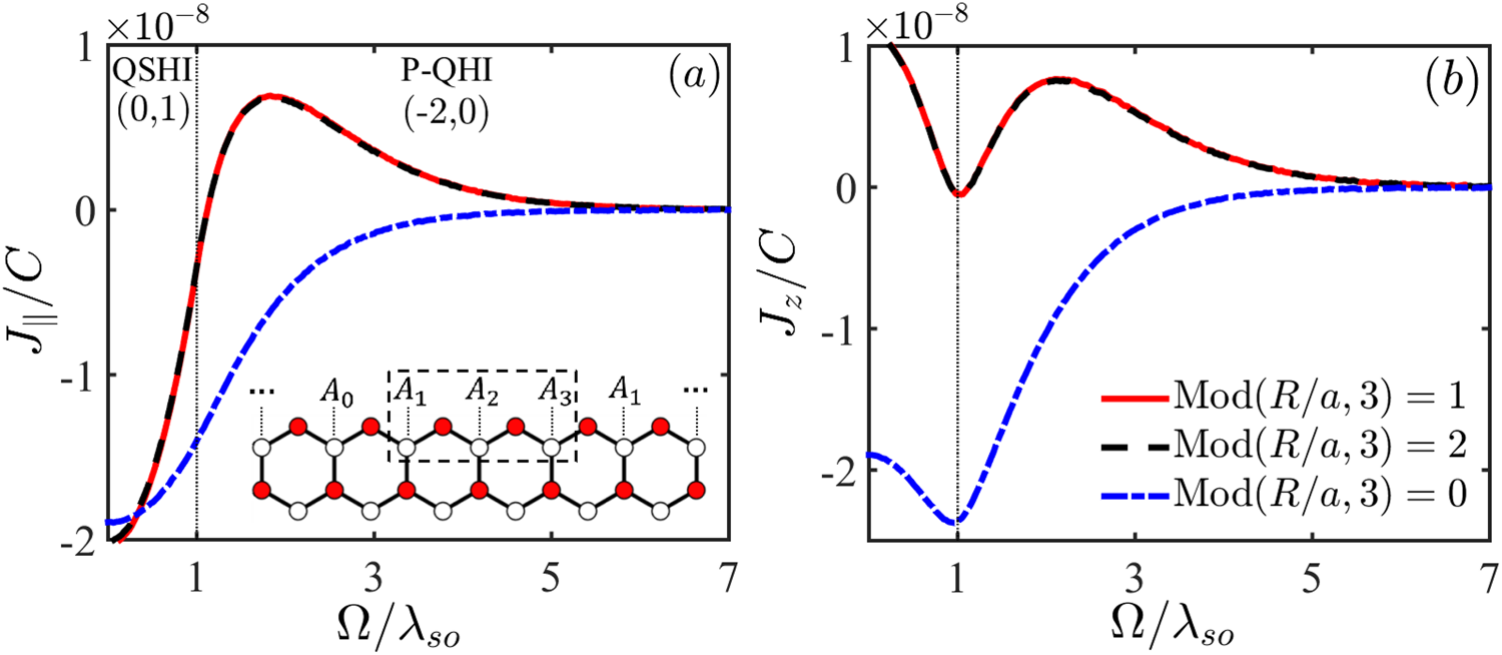
The variation of RKKY exchange coupling with illumination parameter Ω. The QSHI and P-QHI phases are divided by a vertical dotted line. Two impurities are distributed on the same lattice along the zigzag direction, as shown in inset, with three configurations in spatial distance *R* = 270*a* [Mod(*R*/*a*, 3) = 0], 271*a* [Mod(*R*/*a*, 3) = 1] and 272*a* [Mod(*R*/*a*, 3) = 2]. The other parameters are *E*_*F*_ = 0, *t* = 1.6 eV, and *λ*_*so*_ = 3.9 meV.

In Fig. 1, two phase regimes of the QSHI (0, 1) and P-QHI (−2, 0) are divided by a vertical dotted line. In only irradiation of light, the bandgap is reduced to |*V*_*s*_(Ω)|, where the short-hand notation is for *V*_*s*_(*x*) = *x* + *sλ*_*so*_. By substituting the Green’s function Eq. (6) in spin space into Eq. (5) and then taking the matrix trace to cancel the spin degrees of freedom, the RKKY interaction can be written in the form of Eq. (7), where various coefficients are derived as ${J}_{i}=-\,2C{\rm{Im}}\,{\int }_{-\infty }^{{E}_{F}}\,{N}_{i}d\varepsilon$ $(C=8\pi {\lambda }^{2}/{\varsigma }^{2}{\hslash }^{4}{v}_{F}^{4})$ with

8 $${N}_{\parallel }=2[{\varepsilon }^{2}\,{\cos }^{2}\,(\frac{{\bf{1}}}{2}{\rm{\Delta }}{\bf{K}}\cdot {\bf{R}})+{\sin }^{2}\,(\frac{{\bf{1}}}{2}{\rm{\Delta }}{\bf{K}}\cdot {\bf{R}})\,\prod _{s=\pm }\,{V}_{s}({\rm{\Omega }})]\,\prod _{s=\pm }\,{K}_{0}[{ {\mathcal R} }_{{V}_{s}({\rm{\Omega }})}],$$

9 $${N}_{z}=\sum _{s=\pm }\,[{\varepsilon }^{2}\,{\cos }^{2}\,(\frac{{\bf{1}}}{2}{\rm{\Delta }}{\bf{K}}\cdot {\bf{R}})+{\sin }^{2}\,(\frac{{\bf{1}}}{2}{\rm{\Delta }}{\bf{K}}\cdot {\bf{R}})\,{V}_{s}^{2}({\rm{\Omega }})]{K}_{0}^{2}[{ {\mathcal R} }_{{V}_{s}({\rm{\Omega }})}],$$

10 $${N}_{DM}=-\,2{\lambda }_{so}\varepsilon \,\sin ({\rm{\Delta }}{\bf{K}}\cdot {\bf{R}})\,\prod _{s=\pm }\,{K}_{0}[{ {\mathcal R} }_{{V}_{s}({\rm{\Omega }})}],$$

where Δ**K** = **K** − **K**′ is difference of momentum for any two adjacent Dirac points in BZ. We choose two valleys at ${\bf{K}}{\boldsymbol{(}}{\bf{K}}^{\prime} )=\frac{2\pi }{3a}(\,\pm \,1,\sqrt{3})$. Obviously, due to the oscillation factor cos(Δ**K** · **R**) or sin(Δ**K** · **R**), the RKKY interaction is closely related to spatial distance **R** between impurities. While the impurity distance fulfils ${\bf{R}}=na\hat{x}$ along the zigzag direction, the oscillating part sin(*K*_*x*_*R*_*x*_) repeats three values: $\frac{\sqrt{3}}{2}$, −$\frac{\sqrt{3}}{2}$, and 0, corresponding respectively to the impurity configuration satisfied Mod(*R*/*a*, 3) = 1, 2, 0. This is indicated by *A*_1_, *A*_2_ and *A*_3_ in inset of Fig. 1 while the other impurity is fixed at *A*_0_ point. However, sin(Δ**K** · **R)** always vanishes in the armchair direction, making the RKKY featureless, so we in the following focus on the impurities distributed along the zigzag direction and the system is half filled (*E*_*F*_ = 0).

We in Fig. 1 present the numerical results for the illumination dependence of different terms of the RKKY interaction in the long range for three types of impurity positions. For the distances satisfying Mod(*R*/*a*, 3) = 1, 2, there emerges a prominent signature in Fig. 1(a) that the in-plane term ${J}_{\parallel } < 0$ is ferromagnetic in the QSHI phase while it changes to be antiferromagnetic in the P-QHI phase. Interestingly, the transition point is close to the critical value of phase Ω = *λ*_*so*_. This behavior can be understood from Eq. (8), where the second term in ${N}_{\parallel }$ plays a dominant role near the critical point and the sign of its integral is almost determined by ${V}_{+}({\rm{\Omega }}){V}_{-}({\rm{\Omega }})={{\rm{\Omega }}}^{2}-{\lambda }_{so}^{2}$, namely, for QSHI with |Ω| < *λ*_*so*_ the value of ${J}_{\parallel }$ is negative while it is positive otherwise. For the impurity configuration of Mod(*R*/*a*, 3) = 0, no such sign is observable due to sin(Δ**K** · **R/2**) = 0. Besides, it is very interesting to find that the out-plane term *J*_*z*_ in Fig. 1(b) provides more accurate signature of phase transition, manifesting itself by a large dip exactly at the critical point. This dip structure occurs for all of three impurity configurations. In order to understand it, we replace the Bessel function *K*_0_(*x*) with $\sqrt{\pi \mathrm{/2}x}{e}^{-x}$ in the long range^35^ under consideration and taking a derivative of the *N*_*z*_ with respect to Ω. Finally, we obtain a result in the form of $d{N}_{z}/d{\rm{\Omega }}\propto ({\rm{\Omega }}-{\lambda }_{so})f({\rm{\Omega }},\varepsilon )$, which explains the dip feature. Although *J*_*z*_ cannot changes sign like ${J}_{\parallel }$ when the phase transition happens, its magnitude is quantitatively different in QSHI and P-QHI phases. For the DM term *J*_*DM*_, it keeps vanished for the Fermi energy *E*_*F*_ = 0 due to the electron-hole symmetry and the well-preserved inversion symmetry^36^.

**Figure 2:**
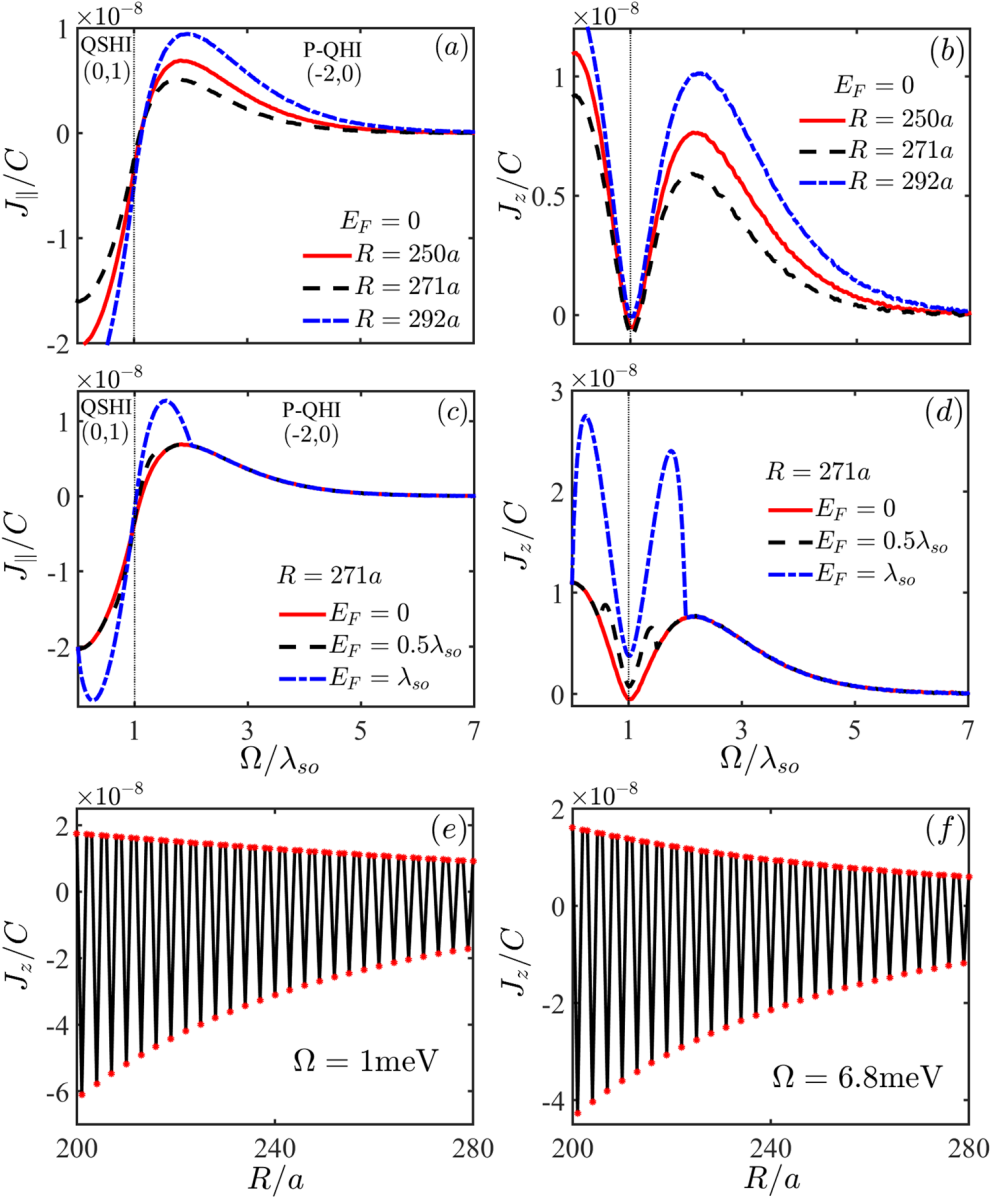
The dependence of ${J}_{\parallel }$ and *J*_*z*_ on Ω for (**a**,**b**) different impurity distances *R* with *E*_*F*_ = 0, (**c**,**d**) for different *E*_*F*_ with *R* = 271*a*. The spatial dependence of ${J}_{\parallel }$ and *J*_*z*_ for (**e**) the QSHI phase (Ω = 1 meV) and (**f**) for the P-QHI phase (Ω = 6.8 meV). The other parameters are the same as in Fig. 1.

In Fig. 2(a,b), we display respectively the in-plane term ${J}_{\parallel }$ and the out-plane term *J*_*z*_ as a function of the illumination parameter Ω. Obviously, the transition point of the ferro-antiferromagnetism remains unchanged for different impurity distance *R* as long as the impurity configuration satisfying Mod(*R*/*a*, 3) = 1 or 2. In the same way, the dip feature in *J*_*z*_ is also independent of the impurity distance *R* though the magnitude of *J*_*z*_ varies in different phases with increase of *R*. These signatures are for *E*_*F*_ = 0. We depict the case for finite Fermi level in Fig. 2(c,d), where the signatures of phase boundary can still keep robust as long as *E*_*F*_ ≤ *λ*_*so*_. If one further increases *E*_*F*_ beyond *λ*_*so*_, the phase boundary becomes fuzzy somewhat, especially for ${J}_{\parallel }$. This can be seen easily from Eq. (8) since the first term cannot be ignored any more with increasing *E*_*F*_. Figure 2(e,f) show the spatial dependence of *J*_*z*_ on the impurity distance *R*, where no difference in the order of magnitude appears in between the QSHI phase [Fig. 2(e)] and P-QHI phase [Fig. 2(d)]. At the same time, one can find the oscillation behavior, which is contributed by an additional phase factor cos(Δ**K** · **R**). Likely, ${J}_{\parallel }$ also presents a similar scenario. The same RKKY oscillations due to the momentum difference Δ**K** of two valleys were in detail discussed in graphene^22^, which is a characteristic of bulk RKKY interaction obtained from the lattice Green’s function. In ref.^16^, this effect is dropped.

### RKKY under electric field

**Figure 3:**
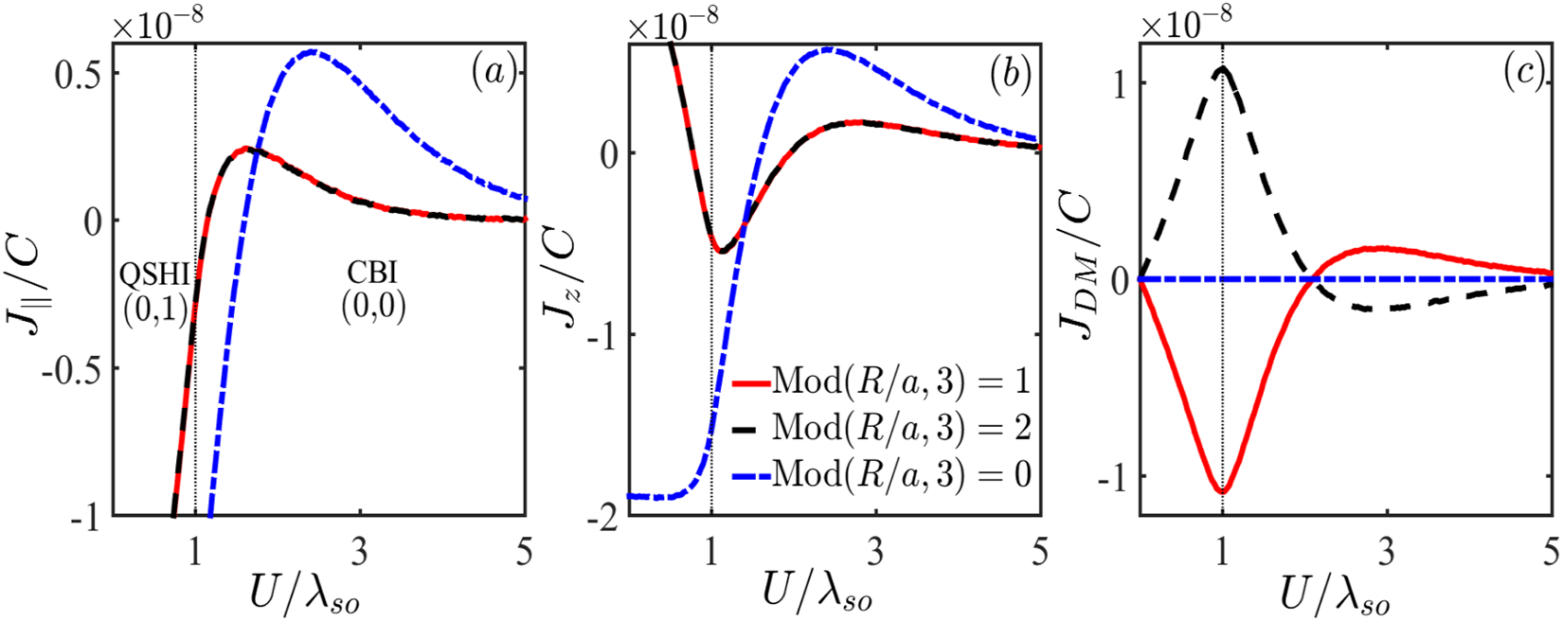
The dependence of (**a**) ${J}_{\parallel }$, (**b**) *J*_*z*_, and (**c**) *J*_*DM*_ on the electric potential *U*. The others are the same as in Fig. 1.

We here discuss the variation of the RKKY interaction when the silicene is subject to a perpendicular electric field *U*. As |*U*| > *λ*_*so*_, the resulting staggered potential can drive the silicene from QSHI phase to CBI phase, whose topological numbers are labeled, respectively, as (0, 1) and (0, 0) in Fig. 3. This topological phase transition is discussed in detail in refs^12,13^. For this case, we derive the RKKY interaction as ${J}_{i}=-\,C{\rm{Im}}\,{\int }_{-\infty }^{{E}_{F}}\,{N}_{i}d\varepsilon$ with

11 $${N}_{\parallel }=2\,\prod _{s=\pm }\,{\zeta }_{s}+\,\cos ({\rm{\Delta }}{\bf{K}}\cdot {\bf{R}})\,\sum _{s=\pm }\,{\zeta }_{s}^{2},$$

12 $${N}_{z}=\sum _{s=\pm }\,{\zeta }_{s}^{2}+2\,\cos ({\rm{\Delta }}{\bf{K}}\cdot {\bf{R}})\,\prod _{s=\pm }\,{\zeta }_{s},$$

13 $${N}_{DM}=\,\sin ({\rm{\Delta }}{\bf{K}}\cdot {\bf{R}})\,\sum _{s}\,s{\zeta }_{s}^{2},$$

where $${\zeta }_{s}=[\varepsilon -{V}_{s}(U)]{K}_{0}[{ {\mathcal R} }_{{V}_{s}(U)}]$$.

Performing the numerical calculations with above expressions, we plot the ${J}_{\parallel }$, *J*_*z*_ and *J*_*DM*_ terms of the exchange coupling in Fig. 3(a–c), respectively. For two impurities placed at Mod(*R*/*a*, 3) = 0, though ${J}_{\parallel }$ and *J*_*z*_ present a transition from the ferromagnetic to antiferromagntic phase, the transition point is far away from the critical point *U* = *λ*_*so*_. In contrast, both ${J}_{\parallel }$ and *J*_*z*_ for impurity configuration Mod(*R*/*a*, 3) = 1, 2 provide a relatively accurate signature for phase boundary: a ferro-to-antiferromagntic transition for ${J}_{\parallel }$ and a dip structure for *J*_*z*_. They are approximately located at the phase transition point. Very different from the case of light irradiation, *J*_*DM*_ shows a strong dependence on the electric field as in Fig. 3(c), where Mod(*R*/*a*, 3) = 1, 2 exhibit a dip and a peak, respectively, providing an unambiguous fingerprint to ascertain the phase boundary between QSHI and CBI. In recent work^16^, the authors found that the RKKY interaction of QSHI phase is about 20 times greater than that in CBI phase, and thus it is proposed to identify the topological phase transition. But, one can notice that the precondition for this signature is that the magnetic impurities must be placed at the edge of silicene where the topological edge states play a crucial role. If the impurities are deposited in bulk, this signature vanishes. On the contrary, in our study the signatures characterizing phase transition stem completely from the bulk band, regardless of the contribution of topological edge states. Thus, one can probe the topological phases simply by measurement of the bulk doping, not needing to elaborately grasp the edge-state contribution, which is experimentally accessible more easily.

### RKKY under both electric and light fields

**Figure 4:**
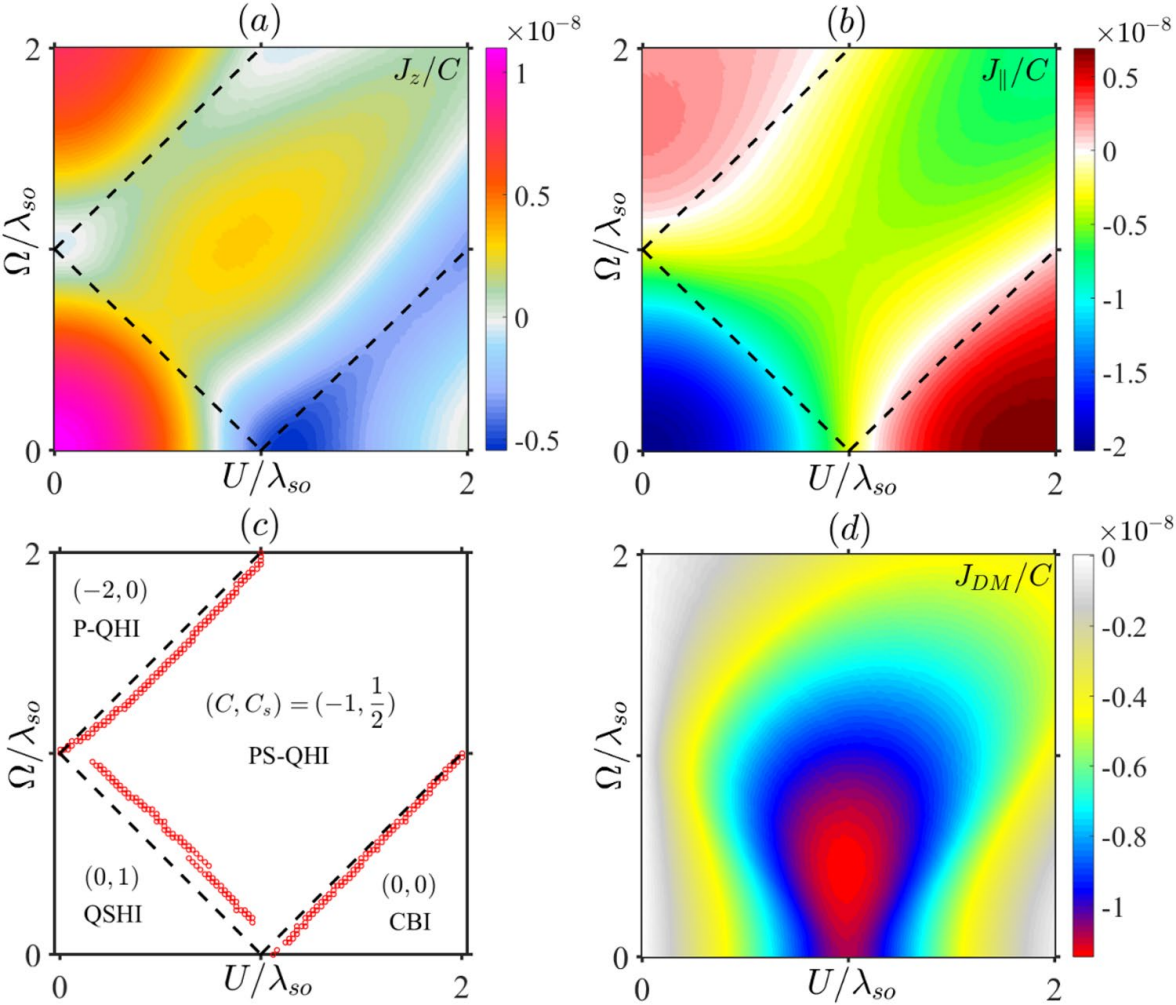
The phase diagrams of (**a**) *J*_*z*_, (**b**) ${J}_{\parallel }$, and (**d**) *J*_*DM*_ as functions of *U* and Ω. (**c**) The comparison between the phase boundary (black dashed lines) and the dip position of *J*_*z*_ (red circles) which is selected from the local minimum value in (**a**). Different phases are labeled by different quantum numbers (*C*, *C*_*s*_), which represent for charge- and spin-Chern numbers, respectively. The chosen parameters are *E*_*F*_ = 0 and *R* = 271*a*.

When both the electric and light fields are exerted, there emerge rich phases: QSHI, P-QHI, PS-QHI, and CBI as shown in Fig. 4(c), where the dashed lines denote the phase boundaries. Since the expressions are too tedious, we here only give the numerical results of *J*_*z*_, ${J}_{\parallel }$, and *J*_*DM*_ for Mod(*R*/*a*, 3) = 1 as functions of the electric potential *U* and illumination parameter Ω in Fig. 4(a,b,d), respectively. Intriguingly, the phase plots in Fig. 4(a,b) present distinct changes in color in different regions, which can be used to differentiate the different phases though it is not too very strict. Importantly, *J*_*z*_ not only has different values for different states, but also clearly characterizes the various phase boundaries, especially for the phase transitions between PS-QHI and CBI, PS-QHI and P-QHI, and QSHI and P-QHI, where a largest dip exists. To compare with the phase plot, we describe the characterizing signatures of the RKKY interaction in Fig. 4(c), marked with red circles by selecting the local minimal values in their boundaries. With a tolerable error, dependence of *J*_*z*_ on electric and light fields provides unambiguous signatures to identify the various phase transitions. By comparison, the phase boundaries of ${J}_{\parallel }$ in Fig. 4(b) become blurry but show remarkable difference in magnitude or sign for different phase regions, suitable for characterizing different phase regions. It is noted that, *J*_*DM*_ in Fig. 4(d) with a deep dip exactly at the critical point can only be applied to divide the phase transition between QSHI and CBI states, but cannot characterize the other intricate phases. As discussed above, the main reason is that *J*_*DM*_ is insensitive to irradiation. Therefore, the measurement of *J*_*z*_ as well as ${J}_{\parallel }$ could be a valid method to divide the different topological areas and their phase boundaries. A recent theoretical work in ref.^14^ proposed that the abundant topological phases can be distinguished by measuring the Nernst conductivity. Notice that there, three types (spin, charge, and valley) of Nernst conductivity have to measure simultaneously, and then to compare them carefully to determine the phase transition boundaries. By contrast, we present an Ising RKKY diagram, from which various phases and their boundaries can be determined simultaneously only by the measurement of one type of the RKKY terms.

## Conclusions

We have studied the RKKY coupling of a monolayer silicene subject to an off-resonant light and a perpendicular electric field. Due to topological phase transition, the RKKY coupling shows strong dependence on the illumination and electric potential. Based on the lattice Green’s function formalism^37^, we have analyzed in detail the variation of the RKKY interaction for different impurity configurations along zigzag direction. It is found that the indirect magnetic interaction has tight connection with various topological phase transitions. For the case irradiated by light, a dip structure of *J*_*z*_ can exactly identify the phase transition of QSHI/P-QHI while the peak or dip of *J*_*DM*_ can feature the critical point of phase transition of QSHI/CBI induced by an electric field. For more complex phase driven by both light and electric fields, it is found that *J*_*z*_ provides information enough to divide the different topological areas with a forgivable error in the phase boundary. Also, ${J}_{\parallel }$ exhibits remarkable difference of magnitude or sign in different phase regions though it is hard to differentiate the phase boundary. Since there are quite rare methods to detect them, especially for the phase transition between PS-QHI and P-QHI, measurement on the RKKY interaction provides us an alternative method to probe the rich topological phases in silicene or other spin-orbit systems. The underlying physics is that both the topological property and magnetic property are determined by bandgap of the band structure. Our proposal is expected to feasible with present technique of spin-polarized scanning tunneling spectroscopy^38^, which can measure the magnetization curves of individual atoms.

## Methods

### Derivation of RKKY interaction

Starting from the effective Hamiltonian ${H^{\prime} }_{\eta s}$ in Eq. (3), the spin- and valley- dependent retarded Green’s function in **k**-space can be calculated as

14 $$\begin{array}{rcl}{G}^{\eta s}({\bf{k}},\varepsilon ) & = & {(\varepsilon +i{0}^{+}-{H^{\prime} }_{\eta s})}^{-1}\\  & = & \frac{1}{{(\varepsilon +i{0}^{+})}^{2}-{\hslash }^{2}{v}_{F}^{2}{k}^{2}-{U}_{\eta s}^{2}}(\begin{array}{cc}\varepsilon +{U}_{\eta s} & \hslash {v}_{F}k{{\rm{\Phi }}}_{{K}_{\eta }}\\ \hslash {v}_{F}k{{\rm{\Phi }}}_{{K}_{\eta }}^{\ast } & \varepsilon -{U}_{\eta s}\end{array}),\end{array}$$

where the matrix is in the sublattice space {*A*, *B*}. Performing a Flourier transformation from **k**-space to real space and only consider the same sublattice *A*,

15 $$\begin{array}{rcl}{G}^{s}({\bf{R}},\varepsilon ) & = & \frac{1}{\varsigma }\,\int \,\sum _{\eta =\pm 1}\,{e}^{i({\bf{k}}+{{\bf{k}}}_{\eta })\cdot {\bf{R}}}{G}^{\eta s}({\bf{k}},\varepsilon ){d}^{2}{\bf{k}}\\  & = & \frac{2\pi }{\varsigma }\,\sum _{\eta =\pm 1}\,{e}^{i{{\bf{K}}}_{\eta }{\bf{R}}}\,{\int }_{0}^{\infty }\,\frac{\varepsilon +{U}_{\eta s}}{{(\varepsilon +i{0}^{+})}^{2}-{\hslash }^{2}{v}_{F}^{2}{k}^{2}-{U}_{\eta s}^{2}}k{J}_{0}(kR)dk\\  & = & -\frac{2\pi }{\varsigma {\hslash }^{2}{v}_{F}^{2}}\,\sum _{\eta =\pm 1}\,{e}^{i{{\bf{K}}}_{\eta }{\bf{R}}}(\varepsilon +{U}_{\eta s}){K}_{0}({ {\mathcal R} }_{{U}_{\eta s}}),\end{array}$$

which is Eq. (6) in maintext. Due to *G*^*s*^(**R**, *ε*) diagonalized in spin space, the RKKY interaction of impurities in Eq. (5) is given by

16 $$\begin{array}{rcl}{H}_{RKKY} & = & \frac{-{\lambda }^{2}}{\pi }\,{\rm{Im}}\,{\int }_{-\infty }^{{E}_{F}}\,{\rm{Tr}}[({{\bf{S}}}_{1}\cdot \sigma )G({\bf{R}},\varepsilon )({{\bf{S}}}_{2}\cdot \sigma )G(\,-{\bf{R}},\varepsilon )]d\varepsilon \\  & = & \frac{-{\lambda }^{2}}{\pi }\,{\rm{Im}}\,{\int }_{-\infty }^{{E}_{F}}\,\sum _{i,j=x,y,z}\,{S}_{1i}{S}_{2j}{\rm{Tr}}[{\sigma }_{i}G({\bf{R}},\varepsilon ){\sigma }_{j}G(\,-{\bf{R}},\varepsilon )]d\varepsilon .\end{array}$$

Inserting the Green’s function *G*(**R**,*ε*) in Eq. (15) into the above equation and calculating the trace of the matrix product *σ*_*i*_*G*(**R**, *ε*)*σ*_*j*_*G*(−**R**, *ε*) over the spin, the RKKY interaction can be divided into three parts

17 $${H}_{RKKY}={J}_{\parallel }\,\sum _{i=x,y}\,{S}_{1i}{S}_{2i}+{J}_{z}{S}_{1z}{S}_{2z}+{J}_{DM}{({{\bf{S}}}_{1}\times {{\bf{S}}}_{2})}_{z},$$

where

18 $${J}_{\parallel }=\frac{-{\lambda }^{2}}{\pi }\,{\rm{Im}}\,{\int }_{-\infty }^{{E}_{F}}\,[{G}_{+}({\bf{R}},\varepsilon ){G}_{-}(\,-\,{\bf{R}},\varepsilon )+{G}_{-}({\bf{R}},\varepsilon ){G}_{+}(\,-\,{\bf{R}},\varepsilon )]d\varepsilon ,$$

19 $${J}_{z}=\frac{-{\lambda }^{2}}{\pi }\,{\rm{Im}}\,{\int }_{-\infty }^{{E}_{F}}\,[{G}_{+}({\bf{R}},\varepsilon ){G}_{+}(\,-\,{\bf{R}},\varepsilon )+{G}_{-}({\bf{R}},\varepsilon ){G}_{-}(\,-\,{\bf{R}},\varepsilon )]d\varepsilon ,$$

20 $${J}_{DM}=\frac{-{\lambda }^{2}}{\pi }\,{\rm{Re}}\,{\int }_{-\infty }^{{E}_{F}}\,[{G}_{-}({\bf{R}},\varepsilon ){G}_{+}(\,-\,{\bf{R}},\varepsilon )-{G}_{+}({\bf{R}},\varepsilon ){G}_{-}(\,-\,{\bf{R}},\varepsilon )]d\varepsilon .$$

For the radiation with light field, *U*_*ηs*_ = *ηV*_*s*_(Ω) = *η*(Ω + *sλ*_*so*_). With the help of Eqs (15) and (18), one can simplify the RKKY component ${J}_{\parallel }$ as

$$\begin{array}{rcl}{J}_{\parallel } & = & -\frac{4\pi {\lambda }^{2}}{{\varsigma }^{2}{\hslash }^{4}{v}_{F}^{4}}\,{\rm{Im}}\,{\int }_{-\infty }^{{E}_{F}}\,[\sum _{\eta ,\eta ^{\prime} =\pm 1}\,{e}^{i({{\bf{K}}}_{\eta }-{{\bf{K}}}_{\eta ^{\prime} }){\bf{R}}}(\varepsilon +\eta {V}_{+})(\varepsilon +\eta ^{\prime} {V}_{-}){K}_{0}({ {\mathcal R} }_{{V}_{+}}){K}_{0}({ {\mathcal R} }_{{V}_{-}})]d\varepsilon \\  &  & -\frac{4\pi {\lambda }^{2}}{{\varsigma }^{2}{\hslash }^{4}{v}_{F}^{4}}\,{\rm{Im}}\,{\int }_{-\infty }^{{E}_{F}}\,[\sum _{\eta ,\eta ^{\prime} =\pm 1}\,{e}^{i({{\bf{K}}}_{\eta }-{{\bf{K}}}_{\eta ^{\prime} }){\bf{R}}}(\varepsilon +\eta {V}_{-})(\varepsilon +\eta ^{\prime} {V}_{+}){K}_{0}({ {\mathcal R} }_{{V}_{-}}){K}_{0}({ {\mathcal R} }_{{V}_{+}})]d\varepsilon \\  & = & -\frac{32\pi {\lambda }^{2}}{{\varsigma }^{2}{\hslash }^{4}{v}_{F}^{4}}\,{\rm{Im}}\,{\int }_{-\infty }^{{E}_{F}}\,[{\varepsilon }^{2}\,{\cos }^{2}\,(\frac{{\bf{1}}}{2}{\rm{\Delta }}{\bf{K}}\cdot {\bf{R}})+{\sin }^{2}\,(\frac{{\bf{1}}}{2}{\rm{\Delta }}{\bf{K}}\cdot {\bf{R}})\,\prod _{s=\pm }\,{V}_{s}({\rm{\Omega }})]\,\prod _{s=\pm }\,{K}_{0}[{ {\mathcal R} }_{{V}_{s}({\rm{\Omega }})}]d\varepsilon .\end{array}$$

We denote ${J}_{i}=-\,2C{\rm{Im}}\,{\int }_{-\infty }^{{E}_{F}}\,{N}_{i}d\varepsilon$ $(C=8\pi {\lambda }^{2}/{\varsigma }^{2}{\hslash }^{4}{v}_{F}^{4})$, and so

21 $${N}_{\parallel }=2[{\varepsilon }^{2}\,{\cos }^{2}\,(\frac{{\bf{1}}}{2}{\rm{\Delta }}{\bf{K}}\cdot {\bf{R}})+{\sin }^{2}\,(\frac{{\bf{1}}}{2}{\rm{\Delta }}{\bf{K}}\cdot {\bf{R}})\,\prod _{s=\pm }\,{V}_{s}({\rm{\Omega }})]\,\prod _{s=\pm }\,{K}_{0}[{ {\mathcal R} }_{{V}_{s}({\rm{\Omega }})}].$$

Similarly, the other RKKY components ${N}_{\parallel }$ and *N*_*DM*_ can be obtained to be Eqs (9) and (10).

In applied electric field where *U*_*ηs*_ = *ηV*_*s*_(*U*) = *U* + *ηsλ*_*so*_, one can perform the same procedure, and the RKKY interaction in Eqs (11–13) are also obtained.
